# Nursing Students and the Human Body: Application of an Ethics Pilot Project

**DOI:** 10.3390/ijerph191811603

**Published:** 2022-09-15

**Authors:** Layla Garrigues, Isabelle Soulé, Amber L. Vermeesch

**Affiliations:** 1 School of Nursing and Health Innovations, The University of Portland, 5000 N, Willamette Blvd., Portland, OR 97203, USA; 2 Department of Family and Community Nursing, School of Nursing, University of North Carolina Greensboro, 1007 Walker Avenue, Greensboro, NC 27402, USA

**Keywords:** nature, connectivity, natural worlds, environment, arts, embodiment, physical body, attunement

## Abstract

This manuscript offers findings from a pilot project which prepares nursing students for embodied professional practice through the lens of ethics. Four undergraduate nursing students were mentored by two nursing faculty in the Dundon-Berchtold Institute Faculty Fellowship Program in the Application of Ethics through an exploration on the ethics of embodiment using an arts pedagogy across one academic year. Inspired by the intersection of nature and health, this project explores the impact of an arts-integrated pedagogy on the human body. The findings from this project provide a natural first step for nursing students to consider multiple interpretations of the human body and to facilitate the students’ development of an embodied ethical practice that is perceptive, empathic, and attuned to themselves as natural beings as well as diverse individuals and populations. The findings from this pilot project presents a pivotal opportunity to guide future nursing curricular development toward holistic, nature-inspired, and mindful-based interventions in order to increase resilience, decrease risk factors of compassion fatigue and burnout, and support nursing students to develop strength-based skills to use in their professional nursing practice.

## 1. Introduction

Nurses have historically and culturally been thought of as bodyworkers as they help to heal the bodies of others using their own bodies as instruments [1,2]. Embodied practice, which refers to the ability to physically experience one’s own and another’s experience, is integral to the nursing profession [3,4,5]; yet there is no mention of this phenomenon within nursing’s main guiding texts on practice [6,7] nor has the concept been robustly examined within nursing education. The purpose of this pilot project was to explore ways to enhance the mind-body awareness of nursing students as a parallel process to refining accurate discernment of the responses of diverse clients and populations. Volunteer participants in this pilot project included four undergraduate nursing students, two upper division and two lower division, ages 18–21 years, in addition to two experienced nursing faculty members. Importantly, this pilot project assumes the relativism of various perspectives, based on the recognition that each of us brings a pre-understanding to a given situation, and that knowledge and meaning are co-created among individuals based on personal, historical and social contexts [8]. Using readings, discussion, and experiential activities, participants examined the concept of embodiment and its role in professional nursing practice.

## 2. Background

Nursing students educated within a Western context likely experience a separation between cognitive and embodied knowledge in favor of cognitive knowing. This practice, often unconscious, brings about nursing care that is enacted as a one-way process, from nurse to client, without recognition of the interconnectedness of perspectives in negotiating care. This way of thinking is a reflection of the Cartesian duality that underpins biomedicine and the mainstream approach to medicine and health care in the United States. Cartesian dualism is a philosophy that states how the body cannot think and that the mind exists by itself outside the body [9]. In the context of nursing education, nursing students are often taught to think about the bodies of others without a concomitant focus on their own multisensory, affective, and behavioral aspects of experience [10]. This Western perspective of the mind-body separation is dominant within both the medical and nursing literature and pedagogy [1,3,11].

**Figure 1:**
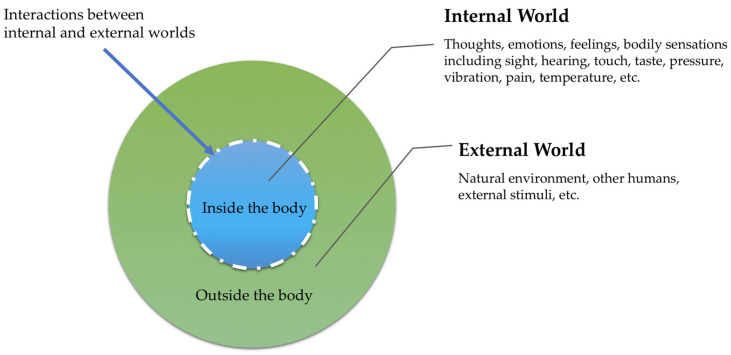
Internal and External Worlds: Nature (Environment).

In this paper, we describe an alternative approach that honors the body and mind equally for their interconnectedness and their unique ways of knowing. There is substantial literature about somatic conditions that are not confined to the mind and additionally, affect the human body, such as post-traumatic stress disorder, metabolic syndromes, and autoimmune conditions [12,13,14]. The human body is in constant interaction with internal and external environments in a complex ecological interaction [15] which we define as part of nature. There is the need for nurses to become aware of, attuned to, and reflect on their own feelings and multisensory awareness that is integral to connected knowing and knowledge. This holistic and integrated approach of embodied practice is vital in order to build interpersonal relatedness and attunement with diverse clients and populations [16,17,18]. In addition, awareness of bodily sensations and responses can help identify early stress cues and create an opportunity to alter one’s inner or outer world to mitigate compassion fatigue and burnout [19]. We define nature as encompassing both these inner and outer worlds (within our human body and external to our human body) that are part of our complex life systems situated in the environment in which we live [15] (see Figure 1). There are continuous, fluid interactions between internal and external worlds depicted by a dashed, permeable line.

Provision 1 of the American Nurses Association (ANA) Code of Ethics for Nurses states that nurses owe the same care for themselves as for others [6]. It is both an ethical duty and moral obligation for nurses to engage in reflective self-care to preserve their own health and well-being [6]. By engaging in experiences that enhance nurses’ awareness of the body’s role in the self, nurses will be better prepared to mindfully care for clients [3,4,20]. An arts-integrated pedagogy is a holistic learning approach that incorporates the intersection between the nature (environment) and the embodied practice of the human body, mind, and emotions [21,22,23].

## 3. Literature Review

The concept of embodiment is described as a way of analyzing, processing, and understanding one’s own and another’s experience [24] through recognition and identification of feelings and bodily sensations [3,4,5]. Embodiment “focuses on the role of the body in shaping our understanding of the world, ourselves, and other people…embodiment describes how we understand and experience the world, other people, and ourselves not simply from the perspective of having or being in a body, but from our perspective as a body” [24] (p. 235). This holistic approach is essential to high-quality nursing practice, and yet nursing education primarily focuses on cognitive knowing, learning skills, and reflection often without an in-depth processing of embodied knowing. In other words, when one of the primary foci of nursing education is cognitive knowing, implicitly the body—including sensations and visceral responses—are excluded as another important way of knowing. This exclusion of the body can lead to a cognitive-based observation of experience rather than the experience itself, alienating us from the complexity of our physical experience including feelings, sensory stimuli, and emotions [11,18,25].

Nurses who become aware of their own internal emotions and visceral responses are better able to interpret those responses, and understand how they can enhance or inhibit building rapport with diverse clients and populations. This blending of alternate ways of knowing allows for a more holistic and ethical approach to caring for clients and communities [3,4]. Because nursing practice relies on developing perceptual acuities to see, hear, feel, and discern patterns (in self and others) often not recognized before professional education, understanding the physical nature of nursing practice can help ease nursing students transition into professional practice [3,26,27].

Furthermore, nursing students are exposed to sensory-intense events in the classroom, simulation, and clinical settings that can cause potential psychological harm. Therefore, it is the ethical responsibility of nursing faculty to create an environment in which nursing students can explore knowledge of the body under safe, protected conditions before entering professional practice. Nurses who have not been guided to consider their own interpretations of the human body or lack awareness of their own sensory responses may be at higher risk of conflicts of conscience, and thus vicarious trauma [28]. The arts and nature-inspired interventions are recommended to improve health and well-being [29,30,31,32,33] and to integrate and embed learning experiences with mindfulness, self-awareness, and stress reduction [3,4,33,34].

## 4. Program Objectives and Goals

The goal of this project was to facilitate the participation of undergraduate nursing students in the pilot project and explore their interpretations of the human body. Several short-term and long-term program goals were identified to guide the development and implementation of the program. The short-term goals included: (a) describe students’ embodiment of nature-inspired experiential learning [29,30,31,32] focusing on the interpretations of the human body through the arts; (b) describe students’ embodied experience of creating an arts-based project to articulate interpretations of the human body; and (c) describe nursing students’ interpretations of the human body that emerge through their artistic presentations. The long-term goals were to (a) develop an elective course on the interpretations of the human body through the arts and (b) embed the exploration of the interpretations of the human body through the arts in the concept-based school of nursing curriculum.

## 5. Materials and Methods

A grant-funded exploratory 15-h workshop-style learning opportunity provided a select group of nursing students the opportunity to explore personal interpretations of the human body via arts-integrated pedagogy. The project was announced in undergraduate nursing classes where students were selected based on their interest in the topic and ability to commit the time and effort in the project. Four undergraduate nursing students and two nursing faculty participated in this exploratory project and acted as pilot recipients and co-creators of this content. All four students worked closely with the two nursing faculty members to create and evaluate activities to increase the interpretations of the human body through nature-inspired arts-pedagogy and made recommendations for integration into the school of nursing curriculum.

This collaborative project required discussion of the power differential between students and faculty and the establishment of a reciprocal learning environment where all group members were considered both teacher and learner. These exploratory sessions were evaluated by both students and faculty for relevance, degree of impact, potential for transformation, and impact on future nursing practice. To address the first short-term goal, to describe students’ embodiment of experiential learning which focus on the interpretation of the human body through the arts, we included specific theoretical and philosophical readings about the human body in addition to using arts-informed pedagogies and activities for students such as: a multi-sensory experience such as storytelling from practice; critiquing photographs; sharing self-reflections; films; nature walks; and art and drawings. To address the second short-term goal, to explore students’ multi-sensory bodily (embodied) experiences as they express their personal interpretations of the body through artistic forms, we coordinated time for the students to develop an arts-based project that represents their personal interpretation of the body. In addition, for the third short-term goal, to explore what interpretation(s) nursing students might attribute to the human body as expressed through their art, we coordinated students’ presentations of their artwork via a public showing of it at the end of the pilot project along with a poster presentation.

Anecdotal notes were kept among students and faculty and a summary of learnings and recommendations were compiled from the project. Students kept journals about how their thinking shifted during reflective activities and in relationship with the group. Instructions from faculty during the sessions included paying attention to the internal sensations of the body, noticing without judgment, slowing down, and assessing how students carried themselves when they walked, and considering different situations and various cultures. They were asked to review what and when they felt triggered, completing body scans and assessing emotional and physical feelings. One purpose for the body scan was to increase self-awareness and to become comfortable, grounded, and “friends with their body”. During their scans they were asked to assess what part of their body they are most uncomfortable with and explore reasons. Students were asked to do one hour in silence, for example, sitting somewhere on campus and observing where many people passed by and to notice people, assessing interactions of others, and their own emotional and physical feelings, assessing intersection of feelings and if there was a sense of resonance or dissonance. Students were asked to “notice” these sensations, feelings, emotions, and thoughts, and not to have any type of judgments such as liking or disliking. During group debriefings students shared and processed these experiences with the group. Another activity involved assessing what students wear privately and publicly, how they felt and trying clothes they were not normally comfortable wearing publicly, and processing thoughts and feelings. Discussion included students’ lived experiences and how their bodies responded differently whether they were wearing casual clothing, nursing scrubs, or uniforms (e.g., Reserve Officers’ Training Corps program uniform).

## 6. Results

### 6.1. Conducting Evaluations

Both process and outcome evaluations were conducted as part of this pilot project. Throughout the sessions, students provided their qualitative feedback in both written and verbal comments, including self-reflections on their learning. In our discussions, we highlighted taken-for-granted conceptualizations of the body as they arose and inquired about the roots of their formation. Examples of these conceptualizations included dominant ideologies of the body within health care such as the artificial conceptual separation of the mind and body, discounting subjective accounts of clients as they relate their lived experiences and perspectives impacting their health and well-being, and the primary medical focus on objective, physiological findings. Developing attention to internal sensory or visceral responses can help nurses in discerning their previously unconscious reactions and how they influence the interactions with others and the environment.

Students processed the challenge of sitting silently in a busy area observing and noticing themselves and others in the area. One student did their observation in the airport where a “mass of bodies” moved through the space and that it became a “collective way rather than individual”. They processed how when they feel uncomfortable they might use distraction such as using electronic devices or working on something, stating, “I feel uncomfortable when I don’t pull out phone when I’m around others with phones”. Another student stated that using electronic devices and accessing social media is “immediate gratification”. Together, students agreed that intentionally putting away their distracting electronic devices, noticing their environment, people, nature, and processing feelings was “eye opening” and “profound”. Students discussed how conducting evaluations took “internal courage” and brought about a sense of “vulnerability” that was both “joyous, freeing,” and “uncomfortable or painful” at the same time. Brené Brown [35] states that “the foundation of courage is vulnerability—the ability to navigate uncertainty, risk, and emotional exposure” (para. 27) and that it also takes courage to feel pain. It was the whole-hearted immersive experiences and their processing that allowed students to strengthen their self-awareness and collective connections.

### 6.2. Awareness Development

Throughout this project, the central focus was on the essential role of embodiment, set a context where deepening self-awareness could occur, explored multiple interpretations of the human body, and created new interpretations of the body that can be contemplated, publicly communicated, advocated for, revitalized, and/or reclaimed within nursing [3,4]. As a result, students who participated in this pilot project described development of their self-awareness and applying their understanding of their interpretations of their physical bodies to work more effectively with diverse individuals and populations.

Some experiential activities included mindful reflections, self-critiques, and watching the movie “Baraka,” a film with no dialogue allowing for personal interpretations and insights [36]. Students engaged in self-reflection and discussion after the movie, exploring their own reactions, feelings, and responses as they tie to the mind-body connection and to the environment surrounding them. Students were asked to focus on reflecting while walking in nature, taking note of any thoughts and bodily sensations they experienced. Students described an increased awareness of the importance of bodywork and multisensory awareness of emotions. They felt a deeper understanding of self and affective responses in different contexts, including between each project session when they were engaging in their classes, colleagues, campus, and community environments. Students stated they had an enhanced ability to perceive, describe, and understand the implications of visceral sensation and how they might be influenced by prior experience or trauma. By the end of the project, students reported having developed a more effective self-care practice in their everyday lives. In addition, the students exhibited an increased sense of inner calm, a recognition of the intersection of personal and professional lives, and personal growth that they described as “profound”. Students stated they wanted this type of engaged learning imbedded into the curriculum and that they could see how this will help them navigate challenging situations and mitigate potential burnout in their nursing practice.

Students discussed how when they suppressed emotions they did not want to feel then it became harder to feel anything, and that suppressing got more challenging with time until it overwhelmed them popping up sporadically and unexpectedly just “like a beach ball that pops up from under the water in strange, unpredictable ways”. Students mused about how to focus only on one of their senses at a time and also which sense they focused on the most. The most common theme was their visual sense and what they saw with their eyes. With focus and self-awareness, students were able to shift their focus from visual senses to hearing sounds and feeling things such as the breeze, heat and cold, these allowed them to process feelings and experiences holistically. Extending these learnings and processing, students discussed about connecting with and noticing clients’ feelings and bodies. Students discussed about the nursing curriculum’s ethical responsibility to prepare nursing students for nursing practice and the importance of integrating learning and processing embodiment using a nature-inspired arts pedagogy. Students discussed about moving away from a “culture competition, comparison, and judgment” and towards “collaboration, cooperation, and compassion for self and others”. Feeling attuned to self, to others, and becoming aware of holistic interactions within and between the inner and external worlds of nature, allowed students to deepen their self-awareness, “feeling more balanced, and compassionate”.

Their expanded understanding of embodied knowing led to a greater capacity for ethical and mindful interactions with others. Students discussed the importance of the integration of ethics and moral obligation and described the sensory experiences as “foundational” to the practice of holistic nursing profession. The students felt it provided them with grounding, strength, and self-reflective practice in assessing themselves for biases, perceptions, and misinterpretations, and monitoring themselves for balance and self-care.

Of particular importance for nursing education, students described the how vital it is to include an ethical orientation to prepare nursing students for professional practice in the 21st century, especially with the increased burdens and stressors of the COVID-19 pandemic and other outbreaks.

## 7. Conclusions

In implementing this reflective learning process, we integrated critical pedagogical practice and focused on the arts, the humanities, and critical theories of the body. The variety of experiential learning activities were used to increase self-awareness of the body and to recognize the effects emotions have on human interactions and the physical environment. Based on the findings of this pilot project, recommendations were made to the undergraduate curriculum committee to include sociologic and symbolic dimensions of the body, and content and learning activities to enhance an understanding of embodied nursing practice. Limitations of this pilot project include a small group size and limited debriefing time of 15 h.

This pilot project provides a first step for nursing students to consider multiple interpretations of the body, and to facilitate their development and growth of an embodied ethical practice that is vital in caring for diverse populations. The findings from this project can help advance the ethical preparedness of new nurses by contributing to curricular review of core curriculum within universities (e.g., ethics courses—philosophy of the body), school of nursing curriculum, (e.g., embodied knowing and vicarious trauma), and transition-to-practice programming. Extending an arts-integrated pedagogy on the human body into the nursing curriculum could begin small with one or two activities in one course such as in an introductory nursing course or a health promotion course, or could be woven into several courses, spiraling in increasing complexity, so that students would be exposed and integrate learning throughout their undergraduate education. This exploratory work will also establish a foundation for further inquiry related to the multiple interpretations of the human body and the interactions between the internal and external worlds of nature from an interdisciplinary perspective. Recommendations for future work include exploring the porosity of the human body as not being separate from the environment [37]. Next steps for expanding this project could be to explore the conceptual shift towards openheartedness [38] and the intra-action and relational connections between and among interactions [37].

The experiential learning activities increased students’ awareness of interactions between body, mind, and the environment. These activities tied in reflecting on any stressful moments or specific challenges students were experiencing at the time and how they go about resolving these sensations, whether mental, psychological, or physical. In addition, these activities increased awareness of the intersection of embodied practice, integrating mental awareness, physical sensations, nature/place-awareness, allowing for more efficacious self-care, wellness, personal growth, grounding, and mindful interactions with others. We recommend further research to explore the multiple interpretations of the human body through experiential learning activities in the undergraduate nursing school curriculum and to better understand the relevance it has to the ethical comportment of professional nursing practice.

## Data Availability

Not applicable.

## References

[1] Marchetti A., Piredda M., De Marinis M.G. (2016). Centrality of Body and Embodiment in Nursing Care: A Scoping Study of the Italian Literature: Body and Embodiment in Nursing. J. Nurs. Scholarsh..

[2] Wolf K.A. (2014). Critical Perspectives on Nursing as Bodywork. Adv. Nurs. Sci..

[3] Harrison H.F., Kinsella E.A., DeLuca S. (2019). Locating the Lived Body in Client–Nurse Interactions: Embodiment, Intersubjectivity and Intercorporeality. Nurs. Philos..

[4] Marchetti A., Piredda M., Facchinetti G., Virgolesi M., Garrino L., Dimonte V., De Marinis M.G. (2019). Nurses’ Experience of Body Nursing Care: A Qualitative Study. Holist. Nurs. Pract..

[5] Mason D.M. (2014). Holism and Embodiment in Nursing: Using Goethean Science to Join 2 Perspectives on Patient Care. Holist. Nurs. Pract..

[6] American Nurses Association (2015). Code of Ethics for Nurses with Interpretive Statements.

[7] (2015). American Nurses Association Nursing: Scope and Standards of Practice.

[8] Heidegger M. (1962). Being and Time.

[9] Damasio A.R. (2005). Descartes’ Error: Emotion, Reason, and the Human Brain..

[10] Draper J. (2014). Embodied Practice: Rediscovering the ‘Heart’ of Nursing. J. Adv. Nurs..

[11] Cussó R.A., González J.S., Murillo D.A., Salgado J.G. (2021). A New Conceptualization of the Nurse–Patient Relationship Construct as Caring Interaction. Nurs. Philos..

[12] Lohr J.B., Palmer B.W., Eidt C.A., Aailaboyina S., Mausbach B.T., Wolkowitz O.M., Thorp S.R., Jeste D.V. (2015). Is Post-Traumatic Stress Disorder Associated with Premature Senescence? A Review of the Literature. Am. J. Geriatr. Psychiatry.

[13] O’Donovan A., Cohen B.E., Seal K.H., Bertenthal D., Margaretten M., Nishimi K., Neylan T.C. (2015). Elevated Risk for Autoimmune Disorders in Iraq and Afghanistan Veterans with Posttraumatic Stress Disorder. Biol. Psychiatry.

[14] Pacella M.L., Hruska B., Delahanty D.L. (2013). The Physical Health Consequences of PTSD and PTSD Symptoms: A Meta-Analytic Review. J. Anxiety Disord..

[15] Dodds J. (2013). Minding the Ecological Body: Neuropsychoanalysis and Ecopsychoanalysis. Front. Psychol..

[16] Dinç L., Gastmans C. (2013). Trust in Nurse–Patient Relationships: A Literature Review. Nurs. Ethics.

[17] Hamington M. (2012). Care Ethics and Corporeal Inquiry in Patient Relations. IJFAB Int. J. Fem. Approaches Bioeth..

[18] Johnson M. (2015). Embodied Understanding. Front. Psychol..

[19] Nolte A.G., Downing C., Temane A., Hastings-Tolsma M. (2017). Compassion Fatigue in Nurses: A Metasynthesis. J. Clin. Nurs..

[20] Vermeesch A.L., Garrigues L., Rothacker-Peyton S. Chapter 18: Rewilding Wellness: An Integrated Perspective. Buttaro Primary Care.

[21] Burnaford G., Brown S., Doherty J., McLaughlin J. (2007). Arts Integration Frameworks, Research, and Practice: A Literature Review. A Literature Review.

[22] Deasy R.J. (2002). Critical Links: Learning in the Arts and Student Academic and Social Development.

[23] Ludwig M., Marklein M.B., Song M. (2016). Arts to Integration: A Promising Approach Improving Early Learning.

[24] Heller T., Harris S.P., Gill C.J., Gould R. (2019). Disability in American Life: An Encyclopedia of Concepts, Policies, and Controversies.

[25] Lakoff G., Johnson M.L. (1999). Philosophy in the Flesh: The Embodied Mind and Its Challenge to Western Thought.

[26] Bennett M.J., Castiglioni I. (2004). Embodied Ethnocentrism and the Feeling of Culture. Handbook of Intercultural Training.

[27] Damasio A.R. (1999). The Feeling of What Happens: Body and Emotion in the Making of Consciousness.

[28] Hartley H. (2018). Concept Review: Second Traumatization and the Role of a Perioperative Advanced Practice Nurse. ORNAC J..

[29] Cooley S.J., Burns V.E., Cumming J. (2015). The Role of Outdoor Adventure Education in Facilitating Groupwork in Higher Education. High Educ..

[30] Dustin D., Furman N., Bricker N., Cederquist J., Schumann S. (2017). The Relevance of Campus Outdoor Recreation Programs to Higher Education: A University of Utah Example. J. Outdoor Recreat. Educ. Leadersh..

[31] Fancourt D., Finn S. (2019). What Is the Evidence on the Role of the Arts in Improving Health and Well-Being? A Scoping Review.

[32] Jordan C., Chawla L. (2019). A Coordinated Research Agenda for Nature-Based Learning. Front. Psychol..

[33] Van Gordon W., Shonin E., Richardson M. (2018). Mindfulness and Nature. Mindfulness.

[34] Caman T. (2008). Chapter 3: Body and World. Merleau-Ponty.

[35] Brown B. Why Experiencing Joy and Pain in a Group Is So Powerful, 2019. https://greatergood.berkeley.edu/article/item/why_experiencing_joy_and_pain_in_a_group_is_so_powerful.

[36] Frick R. (1994). Baraka.

[37] Barad K. (2007). Meeting the Universe Halfway: Quantum Physics and the Entanglement of Matter and Meaning.

[38] Galvin K.T., Todres L. (2009). Embodying Nursing Openheartedness: An Existential Perspective. J. Holist. Nurs..

